# Assessment of Risk and Sero-Prevalence of *Helicobacter pylori* Colonization among Remote Orang Asli Tribes in Peninsula Malaysia

**DOI:** 10.1371/journal.pone.0159830

**Published:** 2016-07-21

**Authors:** Kavitha Thevakumar, Josephine Rebecca Chandren, Guillermo Ignacio Perez-Perez, Eng Guan Chua, Lay Kek Teh, Mohd Zaki Salleh, Jin Ai Mary Anne Tan, Alex Hwong Ruey Leow, Khean Lee Goh, Alfred Chin Yen Tay, Barry J. Marshall, Jamuna Vadivelu, Mun Fai Loke, Li Ping Wong

**Affiliations:** 1 Department of Medical Microbiology, University of Malaya, Kuala Lumpur, Malaysia; 2 Department of Social and Preventive Medicine, Faculty of Medicine, University of Malaya, Kuala Lumpur, Malaysia; 3 Departments of Medicine and Microbiology, New York University School of Medicine and VA Medical Center, New York, United States of America; 4 The Marshall Centre for Infectious Diseases Research and Training, School of Pathology and Laboratory Medicine (M502), University of Western Australia, Perth, Australia; 5 Integrative Pharmacogenomics Institute, Universiti Teknologi MARA, Selangor, Malaysia; 6 Department of Biomedical Science, University of Malaya, Kuala Lumpur, Malaysia; 7 Department of Medicine, University of Malaya, Kuala Lumpur, Malaysia; 8 UM Marshall Centre, High Impact Research Building, University of Malaya, Kuala Lumpur, Malaysia; 9 Julius Centre University of Malaya (JCUM), University of Malaya, Kuala Lumpur, Malaysia; Oita University Faculty of Medicine, JAPAN

## Abstract

The epidemiology of *Helicobacter pylori* (*H*. *pylori*) infection is related to human poverty with marked differences between developing and developed countries. Socioeconomic factors and living standards are the main determinants of the age-dependent acquisition rate of *H*. *pylori*, and consequently its prevalence. The aim of this study was to assess the risk and sero-prevalence of *H*. *pylori* colonization among Orang Asli in Peninsula Malaysia. This cross-sectional study was conducted on Orang Asli subjects in seven isolated settlements spanning across all three major tribes (Negrito, Proto Malay and Senoi) in Malaysia. Socio-demographic characteristics of the subjects were obtained through interview. Subjects were tested for *H*. *pylori* colonization based on CagA and whole cell (WC) antigen serological assays. A total of 275 subjects participated in this study. Among these subjects, 115 (44.7%) were *H*. *pylori* sero-positive with highest sero-prevalence among Negrito (65.7%). Among subjects who were *H*. *pylori* sero-positive, CagA sero positivity was also significantly higher among Negrito. The highest proportion of respondents reported to be *H*. *pylori* sero-positive was from age group 30 years old and below (57.9%), males (56.2%), Negrito (48.6%) and live in bamboo house (92.3%). The highest proportion of respondents reported to be CagA sero-positive was from age group 30 years old and below (41.4%), males (35.6%) and Negrito (48.6%). The results of this study demonstrate that *H*. *pylori* colonization can be related to age, gender, tribes and house materials and CagA sero-positive stain closely associated with age, gender and tribes.

## Introduction

*Helicobacter pylori* (*H*. *pylori*) is a Gram-negative bacillus capable of colonizing the human stomach [1]. More than 50% of the world population is infected with *H*. *pylori*. Prevalence of *H*. *pylori* differs based on geographical, economic and ethnic characteristics of each region [2]. Chronic colonization with *H*. *pylori* is shown to be related to gastric mucosal atrophy, intestinal metaplasia and gastric cancer [1,3,4]. The probable routes of transmission are fecal-oral and oral-oral and thus the risk factors are closely related to food and personal hygiene [5]. Risk factors include low educational level, large number of siblings, household conditions, absence of plumbing system and sanitary facilities, and poor hygiene conditions [6]. *H*. *pylori* colonization may be diagnosed through invasive (rapid urease test, histology and culture of biopsy specimens) and non-invasive techniques (serological tests, fecal antigen assessment and urea breath test) [7]. The serological assessment is considered an acceptable detection technique which may be able to detect past (less than 1 year) and current colonization [7]. Serological assessment determines the presence of Immunoglobulin G (IgG) against *H*. *pylori* [7]. Moreover, serological test may provide additional data on the virulence of the *H*. *pylori* by detecting antibodies against CagA antigen [8].

Malaysia is a country with different ethnicities; among them Malay, Chinese and Indians are the largest sub-populations while Orang Asli (aborigines) comprises only 1% of the population of Malaysia. Three major tribes of Orang Asli are present in Peninsular Malaysia. These tribes include; Negrito, Senoi, and Proto-Malay [9]. The Malaysian government has relocated most of the Orang Asli to periphery of towns with basic education and healthcare facilities. However, most of the Orang Asli remain illiterate and therefore are less aware of personal hygiene and sanitation. The Orang Asli Negrito tribe, also known as Semang, is believed to be the earliest to arrive in Peninsular Malaysia about 25,000 years ago [10,11]. They were separated into six sub-tribes, which are the Kintak, Kensiu, Batek, Mendrik, Jahai, and Lanoh [12]. They are the present day descendants of the early Hoabinhians, who were largely nomadic foragers. However, they now live in permanent settlements in the central, northern and eastern region of Peninsular Malaysia [13]. The Negrito is one of several populations of the seafarer Negrito (which includes Andaman Islanders, the Aeta in Philippines and some Papuan) who are remnants of a previously widely spread Asian population [14]. The Senoi, which include the Mah Meri, Semok Beri, Temiar, Che Wong, Jah Hut, and Semai, reached Peninsular Malaysia during the second wave of migration about 8000 years ago from South Asia, the mountain areas of Cambodia, Vietnam and Burma [12,15]. The Senoi speak Austro-Asiatic languages of the Mon-Khmer sub-group, which reflects their ancient connection with the mainland Southeast Asia [12,13]. However, some believe the Senoi are descendants of Australoid from Australia and Veddoid from South India [16]. The Proto Malays consist of the Jakun, Temuan, Semelai, Kuala, Kanak, and Seletar [17]. They live mainly in the southern part of Peninsular Malaysia and are known to be similar to the Deutero-Malays not only from a morphological standpoint but also culturally and linguistically [11,18]. However, there was evidence of intermarriage and assimilation between this group and the other two earlier groups [14].

The prevalence of *H*. *pylori* colonization was reported to range between 24.3% to 49% in Malaysia as a whole based on histological assessment of samples obtained from endoscopy [4,19,20, 21]. Prevalence of *H*. *pylori* was reported to be higher among Indian (61.8%) and Chinese (48.1%) ethnicities compared to Malay ethnicity (16.4%) [4,19]. Risk factors for *H*. *pylori* colonization among the general Malaysian population includes age (older than 45 years old), Chinese and Indian ethnicities, and low educational level [4,19]. These data are mostly related to the most prevalent ethnicities in Malaysia [4,19,20, 21]. *H*. *pylori* colonization and the related risk factors in the Orang Asli was previously assessed in an earlier study [20]. The risk factors reported were age, sex, occupation, educational level, number of family members in each household, smoking status, alcohol consumption, herbal plants and roots use, source of water supply, diet of exotic foods and family history of gastric disease. In the study, 480 Orang Asli from a settlement in northeastern Malaysia (210 km off Kota Bahru, Kelantan), the prevalence of *H*. *pylori* colonization was reported to be 19% based on serological test for IgG [20]. These findings indicate that *H*. *pylori* colonization does not follow the common national pattern and therefore there is a need for further assessment of the prevalence of *H*. *pylori* colonization and socio-demographic characteristics of the Orang Asli from other settlements. The Orang Asli population in Malaysia is made up of different tribes and sub-tribes, which resides in different geographical regions in the country and with very different lifestyle. In addition, no data exists on the prevalence of virulent *H*. *pylori* strains in the Malaysian Orang Asli population. The main aim of this study was to assess the prevalence of *H*. *pylori* and the prevalence of the more virulent strains (based on CagA sero-positivity) in the Orang Asli population. The second aim was to investigate the association between host socio-demographic characteristics and *H*. *pylori* and CagA IgG sero-positivity.

## Materials and Methods

### Study Subjects

**Fig 1:**
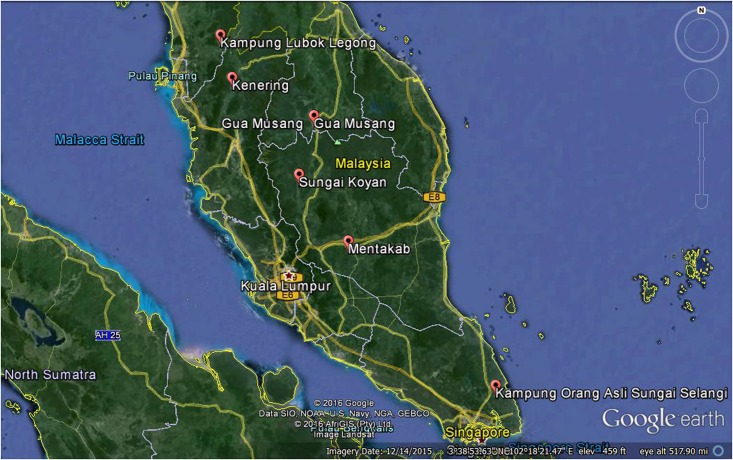
Locations of Orang Asli settlements visited as part of this study. Map of Peninsula Malaysia from Google Earth. Reprinted from Google Earth under a CC BY license, with permission from Google Inc., original copyright 2015.

Seven sub-tribes representing the three indigenous populations from Peninsular Malaysia were included in this study namely, Senoi, Negrito and Proto-Malay (Table 1; Fig 1). A total of 257 samples were recruited for this study. Prior to sample collection, customary visits were made to meet the headmen of the sub-tribes and the community members to explain the nature of the study and to seek their consent. All the households in the sampled indigenous population were invited to participate in the interview survey. Only one member per household was randomly selected to be interviewed. Participant's informed written consent was obtained before commencement of the interview. The interview and the process of obtaining informed consent were conducted in Malay language and witnessed by an accompanied JAKOA officer. Inclusion criteria was participants aged 18 and above and originating from and living in the selected villages.

**Table 1:** Date and location of sampling (N = 257).

Tribes	Sub-tribes	Sampling date	Sampling location	Number of participants
Senoi	Che Wong	30 October– 2 November 2011	Perkampungan Orang Asli Che Wong, Kuala Gandah, Mentakab, Pahang	28
Negrito	Kensiu	17–18 February 2012 and 22–24 November 2012	Perkampungan Orang Asli Kensiu, Lubok Legong, Baling, Kedah	50
Senoi	Semai (Pahang)	16–18 March 2012	Perkampungan Orang Asli Semai, Pos Tual, SG Koyan, Pahang	46
Proto-Malay	Orang Kanaq	2–3 June 2012	Perkampungan Orang Asli Kanaq, SG Selangi, Kota Tinggi, Johor	12
Negrito	Lanoh	24–26 May 2013	Perkampungan Orang Asli Lanoh, Air Bah, Kenering, Perak	34
Negrito	Bateq	27–29 September 2013	Perkampungan Orang Asli Felda Kampung Aring 5, Gua Musang, Kelantan	27
Senoi	Semai (Perak)	-	Perak	60

The questionnaire consist of four mains sections. The first section of the survey consists of socio-demographic characteristics which include educational level, occupation, average household income and type of house. The second section of the questionnaire consists of the activities and lifestyle of participants. This section is divided into three parts which are: a) Contact with domestic or wild animals or insects, b) Personal hygiene and c) Food and nutrition. The third section of the survey consists of questions about gastrointestinal symptoms. The last section consists of family history of *H*. *pylori* colonization.

### *H*. *pylori* Serum Antibody Analysis

A total of eight millilitres of peripheral blood was collected from the participants. Serum IgG was detected by ELISAs, using either *H*. *pylori* whole cell (WC) or CagA protein as antigens. WC antigens were derived from a characterized pool of samples from five US strains. Sensitivity and specificity of this serologic test was 96% and 93.5%, respectively [22,23]. For the WC antigen, an optical density ratio ≥ 1.1 in 1:800 *H*. *pylori* was defined as sero-positive. When the ODR-value was in the range of 0.9 and 1.1 the result was considered equivocal and when the ODR value was <0.9 the result was considered sero-negative.

To assess serologic response to CagA, a 66-kDa *cagA* fragment that had been cloned in *Escherichia coli* as pORV220, was used as antigen [24]. For this assay, serum was diluted 1:100 and was considered sero-positive if the optical density was ≥ 0.35 and a sero-negative result was < 0.30. An equivocal result for CagA was defined when the ODR-value was in the range of 0.3 and 0.35.

### Statistical Analysis

Data was analyzed using IBM SPSS Statistic 21.0 for Windows. The Chi-square was used to test group differences. Bivariate analysis (chi-square test) was conducted to test between socio-demographic with CagA and *H*. *pylori* sero-positivity. Significant predictors of CagA and *H*. *pylori* sero-positivity were assessed using multiple logistic regressions. The significance level was determined at p-value ≤0.05.

### Ethical Statement

This study was reviewed and approved by the Research and Ethics Committee of Universiti Teknologi MARA [Ref no: 600-RMI (5/1/6)] and Department of Orang Asli Development (Jabatan Kemajuan Orang Asli Malaysia, JAKOA) [JHEOA.PP.30.052.JId 5(17)].

## Results

### Socio Demographic Characteristics

A total of 257 participants responded to the questionnaires. Mean age of the subjects was 32.67±12.8 years, ranging from 18 to 73 years, mostly from age group of more than 30 years old (61.5%). There were 101 (39.8%) males and 153 (60.2%) females participated in this study. Most of the subjects were ever married (153, 86.9%) and (23, 13.1%) were single. By tribe, majority were Senoi (134, 52.1%), followed by Negrito (111, 43.2%) and Proto Malay (12, 4.7%). Majority of them (84.3%) have had at least primary education, unemployed (51.4%) and earn RM500 and below as monthly income (86.3%). Most of the respondents were reported to live in detached or single house (99.0%), wooden house (45.9%) with four to six household members (42.5%). The majority used water from government pipe as a source of drinking water (73.7%) and for bathing and washing (61.6%) (Table 2).

**Table 2:** Description of socio demographic data and lifestyle characteristics (N = 257).

Details	Frequency, N (%)
**(A) Socio-demographic data**	
**Age group**	
≤30	99 (38.5)
>30	158 (61.5)
**Gender**^¥^	
Male	101 (39.8)
Female	153 (60.2)
**Marital status**^¥^	
Single	23 (13.1)
Ever Married	153 (86.9)
**Tribe**	
Negrito	111 (43.2)
Proto Malay	12 (4.7)
Senoi	134 (52.1)
**Education**^¥^	
No formal education	54 (38.6)
Primary	64 (45.7)
Secondary	22 (15.7)
**Occupation**^¥^	
Professional & Skilled worker	13 (9.3)
Non-skilled worker	55 (39.3)
Unemployed	72 (51.4)
**Income**^¥^	
≤ RM500	120 (86.3)
> RM500	19 (13.7)
**Household member**^¥^	
1–3	26 (19.4)
4–6	57 (42.5)
7–9	39 (29.1)
≥10	12 (9.0)
**House Type**^¥^	
Terrace/ Link House Primary	1 (1.0)
Detached/ Single House	102 (99.0)
**House material**^¥^	
Bamboo	16 (18.8)
Wood	39 (45.9)
Brick/ Cement	30 (35.3)
**Source of drinking water**^¥^	
Government pipe	101 (73.7)
Well/river	36 (26.3)
**Source of water for bathing and washing**^¥^	
Government pipe	77 (61.6)
Well/river	48 (38.4)
**(B) Lifestyle characteristic**	
**Swimming in river/stream**^¥^	
Never/Rarely	77 (55.4)
Sometimes/ Often	62 (44.6)
**Fishing in river/stream**^¥^	
Never/Rarely	67 (48.2)
Sometimes/ Often	72 (51.8)
**Hunting**^¥^	
Never/Rarely	111 (79.9)
Sometimes/ Often	28 (51.8)
**Smoking history**^¥^	
Ex-smoker	13 (9.4)
Non-smoker	89 (64.0)
Currently smoker	37 (26.6)
**Alcohol consumption**^¥^	
Never/Rarely	137 (98.6)
Sometimes/ Often	2 (1.4)
**Contact with domestic animals:**	
Cat^¥^	
No	85 (61.2)
Yes	14 (38.8)
Dog^¥^	
No	119 (85.6)
Yes	20 (14.4)
Bird^¥^	
No	129 (92.8)
Yes	10 (7.2)
Cow^¥^	
No	140 (100.0)
Yes	-
Goat^¥^	
No	138 (99.3)
Yes	1 (0.7)
Pig ^¥^	
No	138 (99.3)
Yes	1 (0.7)
Chicken/Duck^¥^	
No	68 (48.9)
Yes	71 (51.1)
**Contact with wild animals:**	
Wild pig^¥^	
No	118 (84.9)
Yes	21 (15.1)
Monkey^¥^	
No	120 (86.3)
Yes	7 (15.1)
Wild Bird^¥^	
No	124 (86.3)
Yes	7 (13.7)
**Reporting pest problems in the house:**	
Flies^¥^	
No	74 (53.2)
Yes	65 (46.8)
Cockroaches^¥^	
No	84 (60.4)
Yes	25 (39.6)
Mice/Rodents^¥^	
No	100 (71.9)
Yes	19 (28.1)
**Practices:**	
Boil water before drink^¥^	
Never/Rarely	2 (1.6)
Sometimes/Often	122 (98.4)
Using same glass or cup^¥^	
Never/Rarely	81 (65.3)
Sometimes/Often	43 (34.7)
Using same dish or bowl without washing it first^¥^	
Never/Rarely	91 (73.4)
Sometimes/Often	33 (26.6)
Hand washing before meals^¥^	
Never/Rarely	2 (1.6)
Sometimes/Often	121 (98.4)
Hand washing after toilet use^¥^	
Never/Rarely	2 (1.7)
Sometimes/Often	119 (98.3)
Hand washing with soap^¥^	
Never/Rarely	1 (0.8)
Sometimes/Often	122 (99.2)
Walk barefoot outside the house^¥^	
Never/Rarely	55 (44.7)
Sometimes/Often	68 (55.3)
Eat fiber food^¥^	
Never/Rarely	16 (12.9)
Sometimes/Often	108 (87.1)
Eat red meat^¥^	
Never/Rarely	78 (62.9)
Sometimes/Often	46 (37.1)
Eat poultry products^¥^	
Never/Rarely	43 (34.7)
Sometimes/Often	81 (65.3)
Eat fish/ seafood^¥^	
Never/Rarely	29 (23.4)
Sometimes/Often	82 (76.6)
Eat raw vegetables^¥^	
Never/Rarely	75 (61.0)
Sometimes/Often	48 (39.0)
Eat raw meat^¥^	
Never/Rarely	118 (95.2)
Sometimes/Often	6 (4.8)
Drink milk^¥^	
Never/Rarely	42 (33.9)
Sometimes/Often	82 (66.1)
Drink coffee^¥^	
Never/Rarely	45 (36.3)
Sometimes/Often	79 (63.7)
Drink tea^¥^	
Never/Rarely	26 (21.0)
Sometimes/Often	98 (79.0)

^**¥**^number of respondents less than 257 due to non-response

### Lifestyle Characteristics

Majority of the respondents were never or rarely swimming in river or stream (55.4%) and hunting (79.9%). However, majority of them reported sometimes and often fishing in river or stream (51.8%). Only 26.6% were reported currently smoking and majority never or rarely consumed alcohol (98.6%). In terms of having contact with domestic and wild animals, most of the respondents never have contact with cat (61.2%), dog (85.6%), bird (92.8%), goat (99.3%), pig (99.3%), wild pig (84.9%), monkey (86.3%) and wild bird (86.3%). All of the respondents reported never had contact with cow. However, most of them were reported to ever have contact with chicken and duck (51.1%). Majority of the respondents reported no pests such as flies (53.2%), cockroaches (60.4%) and mice or rodents (71.9%) in the house. Most of the respondent reported that they sometimes or often boiled water before drink (98.4%), wash hands with soap (99.2%), wash hands before meals (98.4%) and after toilet use (98.3%) as well as walk barefoot outside the house (55.3%). In contrast, majority of the respondent reported never or rarely use the same glass or cup (65.3%) and using same dish or bowl without washing it first (73.4%). The majorities reported sometimes or often eat fiber food (87.1%), poultry products (65.3%), fish or seafood (76.6%) as well as drink milk (66.1%), coffee (63.75) and tea (79.0%). On the other hand, the majorities reported never or rarely eat red meat (62.9%), raw meat (95.2%) and raw vegetables (61.0%) (Table 2).

### Characteristics of Gastrointestinal Symptoms

When the respondents were asked about having experienced gastrointestinal symptoms, most of the respondents reported that they never or rarely experienced abdominal pain or unpleasant sensation (77.7%), prolonged persistence of food in stomach (79.1%), feeling of fullness after starting to eat (71.2%), burning sensation (81.3%), reflux or regurgitation (82.6%), upper abdominal bloating (79.0%) and nausea (88.5%) (Table 3).

**Table 3:** Description of gastrointestinal symptoms.

Gastrointestinal symptoms	Frequency, N (%)
**Abdominal pain or unpleasant sensation**^¥^	
Never/Rarely	108 (77.7)
Sometimes/Often	31 (22.3)
**Prolonged persistence of food in stomach**^¥^	
Never/Rarely	110(79.1)
Sometimes/Often	29 (20.9)
**Feeling of fullness after starting to eat**^¥^	
Never/Rarely	99 (71.2)
Sometimes/Often	40 (28.8)
**Burning sensation**^¥^	
Never/Rarely	113 (81.3)
Sometimes/Often	26 (18.7)
**Reflux/Regurgitation**^¥^	
Never/Rarely	114 (82.6)
Sometimes/Often	24 (17.4)
**Upper Abdominal Bloating**^¥^	
Never/Rarely	109 (79.0)
Sometimes/Often	29 (21.0)
**Nausea**^¥^	
Never/Rarely	123 (88.5)
Sometimes/Often	16 (11.5)

^**¥**^number of respondents less than 257 due to non-response

### *H*. *pylori* and CagA Serology

*H*. *pylori* serology tests were carried out on the serum samples of 257 subjects (134 Senoi, 111 Negrito and 12 Proto-Malay) as shown in Table 4. Among these subjects, 115 (44.7%) were found to be either positive for anti-WC and/or anti-CagA. In contrast, only 86 (33.5%) subjects were sero-positive for anti-WC and 72 (28%) were sero-positive for anti-CagA. Only 43 (16.7%) subjects were positive for both anti-WC and anti-CagA while 72 (28.0%) were positive for either anti-WC or anti-CagA. *H*. *pylori* sero-positivity was significantly higher among Negrito compared to Senoi (p-value <0.001). Among subjects who were *H*. *pylori* sero-positive, CagA sero-positivity was significantly higher among Negrito compared to Proto Malay and Senoi (p-value = 0.016 and 0.02).

**Table 4:** *H*. *pylori* and CagA status (n = 257).

	*Frequency*, *N (%)*				*Fisher’s exact test*		
	*Negrito*	*Proto Malay*	*Senoi*	*Total*	*Negrito vs*. *Proto Malay*	*Negrito vs*. *Senoi*	*Proto Malay vs*. *Senoi*
***Anti-WC***							
Sero-positive	54 (48.6)	6 (50)	26 (19.4)	86 (33.5)			
Sero-negative	54 (48.6)	5 (41.7)	96 (71.6)	155 (60.3)	0.423	<0.001**	0.041*
Equivocal	3 (2.7)	1 (8.3)	12 (9)	16 (6.2)			
***Anti-CagA***							
Sero-positive	54 (48.6)	1 (8.3)	17 (12.7)	72 (28)	0.012*	<0.001**	1.000
Sero-negative	57 (51.4)	11 (91.7)	117 (87.3)	185 (72)			
***Anti-WC + Anti-CagA***							
Sero-positive	35 (31.5)	0	8 (6)	43 (16.7)	0.019*	<0.001**	1.000
Sero-negative	36 (32.4)	5 (41.7)	88 (65.7)	129 (50.2)			
***Overall H*. *pylori status***							
Sero-positive	73 (65.8)	7 (58.3)	35 (26.1)	115 (44.7)			
Sero-negative	36 (32.4)	5 (41.7)	88 (65.7)	129 (50.2)	0.624	<0.001**	0.081
Equivocal^¥^	2 (1.8)	0	11 (8.2)	13 (5.1)			
Total	111	12	134	257			
***CagA-positive H*. *pylori***	54 (73.8)	1 (14.3)	17 (48.6)	72 (62.6)	0.016*	0.020*	0.312

*significant at level p<0.05

**significant at level p<0.01

^**¥**^The respondent with equivocal status of *H*. *pylori* was taken out due to small group size.

### Association between *H*. *pylori* Sero-Positivity with Socio Demographic, Lifestyle Characteristics and Gastrointestinal Symptoms

Univariate analysis showed that *H*. *pylori* sero-positivity exhibited significant differences across age, gender, tribes and house material. The respondent with equivocal status of *H*. *pylori* was taken out due to small group size. There were no statistical significant differences between *H*. *pylori* sero-positivity and all gastrointestinal symptoms variables. The highest proportion of respondents reported to be *H*. *pylori* sero-positive was from age group 30 years old and below (57.9%), males (56.2%), Negrito (67.0%) and live in bamboo house (92.3%).

Multivariate logistic regression analysis revealed respondent who lived in wooden house (OR = 0.062, 95% CI, 0.006–0.603, p<0.05) has significantly smaller likelihood to being diagnosed as *H*. *pylori* sero-positive compared to those with a reference level of bamboo type of house. It is noted that all 16 respondents who lived in bamboo houses were of Senoi origin. Among respondents living in bamboo houses, 12 (75.0%) were *H*. *pylori* sero-positive, 3 (18.8%) with equivocal and only 1 (6.3%) was sero-negative. Multivariate logistic regression analysis revealed Senoi respondent who lived in bamboo house (OR = 2.996, 95% CI, 1.655–241.723, p<0.05) has significantly higher likelihood of being diagnosed as *H*. *pylori* sero-positive compared to those with a reference level of brick or cement house. Respondent who lived in the brick or cement house also has smaller likelihood (OR = 0.070, 95% CI, 0.009–1.012) to being diagnosed as *H*. *pylori* sero-positive compared with those with a reference level of bamboo type of house. However, this finding is not statistically significant.

Respondents with age group of more than 30 years old (OR = 0.588, 95% CI, 0.205–1.686) were less likely to being diagnosed as *H*. *pylori* sero-positive compared to with those of reference level of age group of 30 years old and below. Women respondents were more likely (OR = 1.811, 95% CI, 0.503–6.523) to being diagnosed as *H*. *pylori* sero-positive compared with those of reference level of male. Negrito tribe (OR = 1.036, 95% CI, 0.306–3.503) and Proto Malay tribe were more likely (OR = 2.010, 95% CI, 0.329–12.257) to being diagnosed as *H*. *pylori* sero-positive compared with those of reference level of Senoi tribe. However, these findings were not statistically significant (Table 5).

**Table 5:** tatistical analysis of relationship of *H*. *pylori* sero-positivity with socio-demographic data.

Details	Frequency, N (%)	*H*. *pylori* status (n = 244)			Multiple logistic regression for sero-positive vs sero-negative
		Sero-positive	Sero-negative	p-value	Adjusted OR (95% CI)
**Age group**					
≤30	95 (38.9)	55 (57.9)	40 (42.1		Reference
>30	149 (61.1)	60 (40.3)	89 (59.7	**0.007**	0.588 (0.205–1.686)
**Gender**^¥^					
Male	96 (39.8)	54 (56.2)	42 (43.8)		Reference
Female	145 (60.2)	60 (41.4)	85 (58.6)	**0.026**	1.811 (0.503–6.523)
**Tribes**					
Negrito	109 (44.7)	73 (67.0)	36 (33.0)		1.036 (0.306–3.503)
Proto Malay	12 (4.9)	7 (58.3)	5 (41.7)	**0.000**	2.010 (0.329–12.257)
Senoi	123 (50.4)	35 (28.5)	88 (71.5)		Reference
**House material** ^¥^					
Bamboo	13 (16.9)	12 (92.3)	1 (7.7)		Reference
Wood	36 (46.8)	17 (47.2)	19 (52.8)	**0.018**	0.062 (0.006–0.603)*
Brick/ Cement	28 (36.4)	17 (60.7)	11 (39.3)		0.070 (0.009–1.012)

^**¥**^number of respondents less than 244 due to non-response

*significant at level p<0.05

### Association between CagA Sero-Positivity with Socio Demographic, Lifestyle Characteristics and Gastrointestinal Symptoms

Univariate analysis showed that CagA sero-positivity exhibited significant differences across age, gender and tribes. There were no statistical significant differences between CagA sero-positivity and lifestyle characteristics variables and gastrointestinal symptoms variables. The highest proportion of respondents reported to be CagA sero-positive was from age group 30 years old and below (41.4%), males (35.6%) and Negrito (48.6%).

Multivariate logistic regression analysis revealed age group of more than 30 years old (OR = 0.472, 95% CI, 0.255–0.873, p<0.05) significantly were less likely to be CagA sero-positive compared to with those of reference level of age group of 30 years old and below. Negrito tribe was significantly more likely (OR = 5.580, 95% CI, 2.926–10.639, p<0.01) to be CagA sero-positive compared with those of reference level of Senoi tribe.

In the other hand, Proto Malay tribe was less likely (OR = 0.802, 95% CI, 0.094–6.815) to be CagA sero-positive as compared with those of reference level of Senoi tribe. Female respondent were less likely (OR = 0.687, 95% CI, 0.372–1.270) to be CagA sero-positive compared with those of reference level of male. However, there are no statistical significant differences for these findings (Table 6).

**Table 6:** Statistical analysis of relationship of CagA sero-positivity with socio demographic data.

Details	Frequency, N (%)	CagA Status (N = 257)			Multiple logistic regression for sero-positive vs. sero-negative
		Sero-positive	Sero-negative	p-value	Adjusted OR (95% CI)
**Age group**					
≤30	99 (38.5)	41 (41.4)	58 (58.6)		Reference
>30	158 (51.8)	31 (19.6)	127 (80.4)	**0.000**	0.472 (0.255–0.873)*
**Gender**^¥^					
Male	101 (39.8)	36 (35.6)	65 (64.4)		Reference
Female	153 (60.2)	35 (22.9)	118 (77.1)	**0.032**	0.687 (0.372–1.270)
**Tribes**					
Negrito	111 (43.2)	54 (48.6)	57 (51.4)		5.580 (2.926–10.639)**
Proto Malay	12 (4.7)	1 (8.3)	11 (91.7)	**0.000**	0.802 (0.094–6.815)
Senoi	134 (52.1)	17 (12.7)	117 (87.3)		Reference

^**¥**^number of respondents less than 257 due to non-response

*significant at level p<0.05

**significant at level p<0.01

## Discussion

This study provided us the opportunity to investigate the risk factors and prevalence rate associated with *H*. *pylori* colonization among Orang Asli tribes residing in less accessible areas of Peninsular Malaysia. Many of these villages were only accessible by four-wheel drive vehicles and without stable electrical supplies. Thus, serology-based assays were chosen to investigate the extent of exposure to *H*. *pylori* using sera collected from Orang Asli living in remote villages.

Whole cell antigen used in our serology test was prepared from a mix of five US *H*. *pylori* strains and previous studies have shown that antigenic differences exist among the *H*. *pylori* strains from different geographical regions [24,25]. To overcome the problem of poor antigenic recognition, two different antigens (WC and CagA) were used in this study. In a previous study in Ladakh, it was shown that as much as 27% of *H*. *pylori* culture-positive subjects responded to only the CagA antigen but not the WC antigen [24,25]. Thus, in this study, positive for WC and/ or CagA serology test was taken as *H*. *pylori* sero-positive.

Univariate analysis found that *H*. *pylori* sero-positive prevalence was higher among respondents who lived in bamboo house. In the multivariate analysis, house material is also found to be a significant predictor for *H*. *pylori*, where Senoi respondents who lived in bamboo house were at higher risk of being infected with *H*. *pylori*. To date, most Orang Asli has been relocated to the better settlement with better amenities and facilities by the government [26]. The groups who still lived in the bamboo house can be considered as primitive groups where they lived in deep of the jungle or very remote areas. Unpublished finding from a study done on Orang Asli in Peninsular Malaysia shows that majority of Orang Asli who lived in the bamboo house used well and river water for drinking, washing and bathing, which led them to be at higher risk of being infected with *H*. *pylori*.

A significantly higher proportion of males than females were *H*. *pylori* sero-positive. Majority of the males respondents in this study were smokers. Smoking is reported to cause an increase in acid and pepsin secretion and changes in gastric motility, prostaglandin synthesis, gastric mucosal blood flow and mucus secretion [27].

Various studies have reported that the prevalence of *H*. *pylori* colonization increases with age [28,29,30]. In this study, the likelihood of being diagnosed as *H*. *pylori* sero-positive was higher for respondents aged 30 years old or below, however the association is not statistical significance. Nevertheless, our study found significant association age and CagA status. Further, participants of older age group who were *H*. *pylori* sero-positive were significant less likely to be CagA sero-positive compared to the younger age group. This finding was attributable perhaps to a change in *H*. *pylori* population involved in more recent incidents of colonization. The *cagA*- strains that had colonized older Orang Asli above 30 years old in the past may less virulent than the *cagA*+ strains that are colonizing the younger Orang Asli now. Unfortunately, due to limited medical facilities and equipment’s on-site, it was not possible to perform endoscopic examination and to collect gastric biopsy samples for *H*. *pylori* culturing and genotyping.

In this study, *H*. *pylori* and CagA sero-positivity were found to be significantly higher in Negrito compared to Proto Malay and Senoi. The difference between the lifestyle and practices may contribute to the difference in *H*. *pylori* sero-prevalence. Likewise, tribes was also significantly associated to CagA status. Negrito have significantly greater likelihood of being colonized with *cagA*+ strain compared to Senoi and Proto Malay. *cagA+* strains were found in 74.0% Negrito who were *H*. *pylori* sero-positive. According to Tan et al (2005), *cagA*+ strains are the predominant genotypes in all ethnic groups in Malaysia; Malay, Chinese and Indian [31]. Similarly, the sero-prevalence of *H*. *pylori* among asympomtatic volunteers from highly urbanized Kuala Lumpur was 38.6% (91/236) and among those positive for *H*. *pylori* by serology, 83.5% (76/91) were sero-positive for CagA (data not shown). This lead us to hypothesize that the local aborigines had low prevalence of *H*. *pylori* and were colonized mainly with the less virulent *cagA*- strains when they first settled in this part of the world. This view is further supported by the sequencing of the Sahul64, a *H*. *pylori* strain isolated from an indigenous Australian, which lacks the *cag*PAI and posseses a nontransportable VacA protein [32]. Negrito have been known to interact with Malays who resided along the fringes of the forest [26]. Hence, the recent increase in colonization among the Orang Asli (especially Negrito) may have arrived through recent culture integration.

It is worth noting that two separate trips were made to visit the same Kensiu village of the Negrito tribe a year apart. Two subjects whose serums were sero-negative during the first sampling were tested positive for the second sample. This indicated active transmission of *H*. *pylori* in the village. Co-incidentally, both subjects were 23 and 25 years of age. Despite the small sample size to be statistically significant, this finding serves as a warning that *H*. *pylori* colonization may be on the rise in this village.

The limitations of the present study were the relatively small number of respondents. This is due to logistic difficulties of communication, transportation and access to these Orang Asli villages. However, all possible efforts have been made in order to get as many household as possible to be interviewed. This study is also based on the self-reported prevalence which may cause report bias.

## Conclusion

This study revealed that the sero-prevalence of *H*. *pylori* colonization in Malaysian Orang Asli tribes (especially Negrito) was higher than previous report. Moreover, a significant relationship was found between *H*. *pylori* sero-positivity and age, gender, tribe and building material. CagA sero-positivity was significantly associated with age, gender and tribe.
